# The Cancer Epitope Database and Analysis Resource: A Blueprint for the Establishment of a New Bioinformatics Resource for Use by the Cancer Immunology Community

**DOI:** 10.3389/fimmu.2021.735609

**Published:** 2021-08-24

**Authors:** Zeynep Koşaloğlu-Yalçın, Nina Blazeska, Hannah Carter, Morten Nielsen, Ezra Cohen, Donald Kufe, Jose Conejo-Garcia, Paul Robbins, Stephen P. Schoenberger, Bjoern Peters, Alessandro Sette

**Affiliations:** ^1^Center for Infectious Disease and Vaccine Research, La Jolla Institute for Immunology, La Jolla, CA, United States; ^2^Department of Medicine, University of California San Diego, La Jolla, CA, United States; ^3^Moore’s Cancer Center, University of California San Diego, La Jolla, CA, United States; ^4^Department of Bio and Health Informatics, Technical University of Denmark, Lyngby, Denmark; ^5^Instituto de Investigaciones Biotecnológicas, Universidad Nacional de San Martín, San Martín, Argentina; ^6^Dana Farber Cancer Institute, Harvard Medical School, Boston, MA, United States; ^7^Department of Gynecologic Oncology, H. Lee Moffitt Cancer Center and Research Institute, Tampa, FL, United States; ^8^Department of Immunology, H. Lee Moffitt Cancer Center and Research Institute, Tampa, FL, United States; ^9^National Cancer Institute, National Institutes of Health, Bethesda, MD, United States; ^10^Laboratory of Cellular Immunology, La Jolla Institute for Immunology, La Jolla, CA, United States

**Keywords:** cancer, epitope analysis, database (all types), neoantigen, bioinformatics

## Abstract

Recent years have witnessed a dramatic rise in interest towards cancer epitopes in general and particularly neoepitopes, antigens that are encoded by somatic mutations that arise as a consequence of tumorigenesis. There is also an interest in the specific T cell and B cell receptors recognizing these epitopes, as they have therapeutic applications. They can also aid in basic studies to infer the specificity of T cells or B cells characterized in bulk and single-cell sequencing data. The resurgence of interest in T cell and B cell epitopes emphasizes the need to catalog all cancer epitope-related data linked to the biological, immunological, and clinical contexts, and most importantly, making this information freely available to the scientific community in a user-friendly format. In parallel, there is also a need to develop resources for epitope prediction and analysis tools that provide researchers access to predictive strategies and provide objective evaluations of their performance. For example, such tools should enable researchers to identify epitopes that can be effectively used for immunotherapy or in defining biomarkers to predict the outcome of checkpoint blockade therapies. We present here a detailed vision, blueprint, and work plan for the development of a new resource, the **C**ancer **E**pitope **D**atabase and **A**nalysis **R**esource (CEDAR). CEDAR will provide a freely accessible, comprehensive collection of cancer epitope and receptor data curated from the literature and provide easily accessible epitope and T cell/B cell target prediction and analysis tools. The curated cancer epitope data will provide a transparent benchmark dataset that can be used to assess how well prediction tools perform and to develop new prediction tools relevant to the cancer research community.

## Introduction

Recent years have witnessed a dramatic rise in interest towards cancer epitopes, studies that have been greatly facilitated by the dramatic decrease in the cost of whole-exome and transcriptome sequencing, as well as advances in mass spectrometry that has resulted in the generation of large datasets of candidate T cell epitopes that are naturally processed and presented (1). This resurgence of interest is linked to the exceptional success of immune checkpoint blockade therapies that disengage immune suppressive mechanisms and enable cancer antigen-specific T cells to recognize and attack tumor cells expressing those antigens (2–4). Additionally, current research suggests that combining checkpoint blockade treatment and neoantigen-directed therapies, such as vaccines or adoptive T cell transfer, can enhance treatment efficacy (5). More recently, checkpoint blockade therapies have been expanded to the neoadjuvant pre-surgical setting, where the aim is to enhance systemic immunity against a broader set of tumor antigens to eliminate micro-metastatic tumors that would otherwise be the source of a relapse (6). Despite these advances, only a subset of patients benefits from these immunotherapies.

Comprehensively cataloging all cancer epitope-related data linked to the biological, immunological, and clinical contexts will aid in understanding the biological mechanisms associated with efficacy and developing more effective therapeutic approaches. In parallel, researchers need access to computational epitope prediction and analysis tools but also need resources to aid in objective evaluation of the performance of different predictive strategies.

There have been several recent efforts to address these needs. The TANTIGEN 2.0 database (7) contains curated epitope and ligand elution data for many different cancer antigens, such as neoantigens and differentiation antigens. However, TANTIGEN does not include peptides that were shown to be ineffective and also lacks any association with clinical data. Similarly, The Cancer Antigenic Peptide Database (https://caped.icp.ucl.ac.be) also only includes curated epitope data for several different cancer antigens. NEPdb (8) contains curated neoantigens but lacks any other types of cancer antigens. For cataloged neoepitopes, associated receptor information and clinical data are also provided if available. It is possible to query NEPdb for an epitope sequence of interest, but there is no option to search for receptors. dbPepNeo (9) only contains curated HLA class I restricted neoantigens and ligand elution data. Importantly, while all resources provide some basic tools to query the databases for cancer types and peptide sequences, it is not possible to perform specific and granular queries. These resources do also not allow the user to perform any predictions for peptides of interest.

To fill these gaps, we here describe the plans and blueprint to develop a new resource, the Cancer Epitope Database and Analysis Resource (CEDAR). CEDAR is envisioned as a comprehensive bioinformatics resource, which will provide access to curated cancer epitope data, including mutated and non-mutated cancer epitopes, and bioinformatics tools for epitope and receptor analysis and prediction. The work proposed here will build on the Immune Epitope Database (IEDB), in existence since 2003, fully operational and independently funded until at least 2025 by the National Institute of Allergy and Infectious Diseases (NIAID) (10). The IEDB’s focus is on allergy, infectious disease, transplantation, and autoimmunity but does not include cancer. Analogous to the IEDB, CEDAR will include all cancer-specific epitope data from various T cell and B cell experiments, MHC binding assays, and MHC ligandomics by mass-spectrometry. CEDAR will also include results from *in vivo* experiments such as tumor rejection and/or tumor control data. The granular curation of the data and the flexible query structure of CEDAR will allow the user to perform detailed queries to retrieve epitopes supported by different experimental data.

We believe that CEDAR will address an existing need because there is currently no comprehensive informatics resource available to the scientific community that stores data on cancer epitopes, the receptors that recognize them, and the immunological, clinical, and biological context in which they are recognized. In addition to a database of cancer epitopes, CEDAR will provide a set of analysis and prediction tools that will enable cancer researchers to predict putative epitope targets in a tumor sample of interest and also predict the likely specificity of T cell receptors (TCR) or B cell receptors (BCR) identified in single-cell sequencing data. CEDAR will also include benchmarking of existing epitope prediction tools and provide side-by-side comparisons of performance. The significance of these features lies in their utility for the broader community of cancer researchers. Currently, many cancer researchers are using the IEDB and its related tools to attempt to answer questions like this, which is suboptimal given that the IEDB was designed for applications outside of cancer (11).

## Results

### A Plan to Define the CEDAR Database Scope Based on the Salient Characteristics of Cancer Epitope Data and Metadata

**Figure 1:**
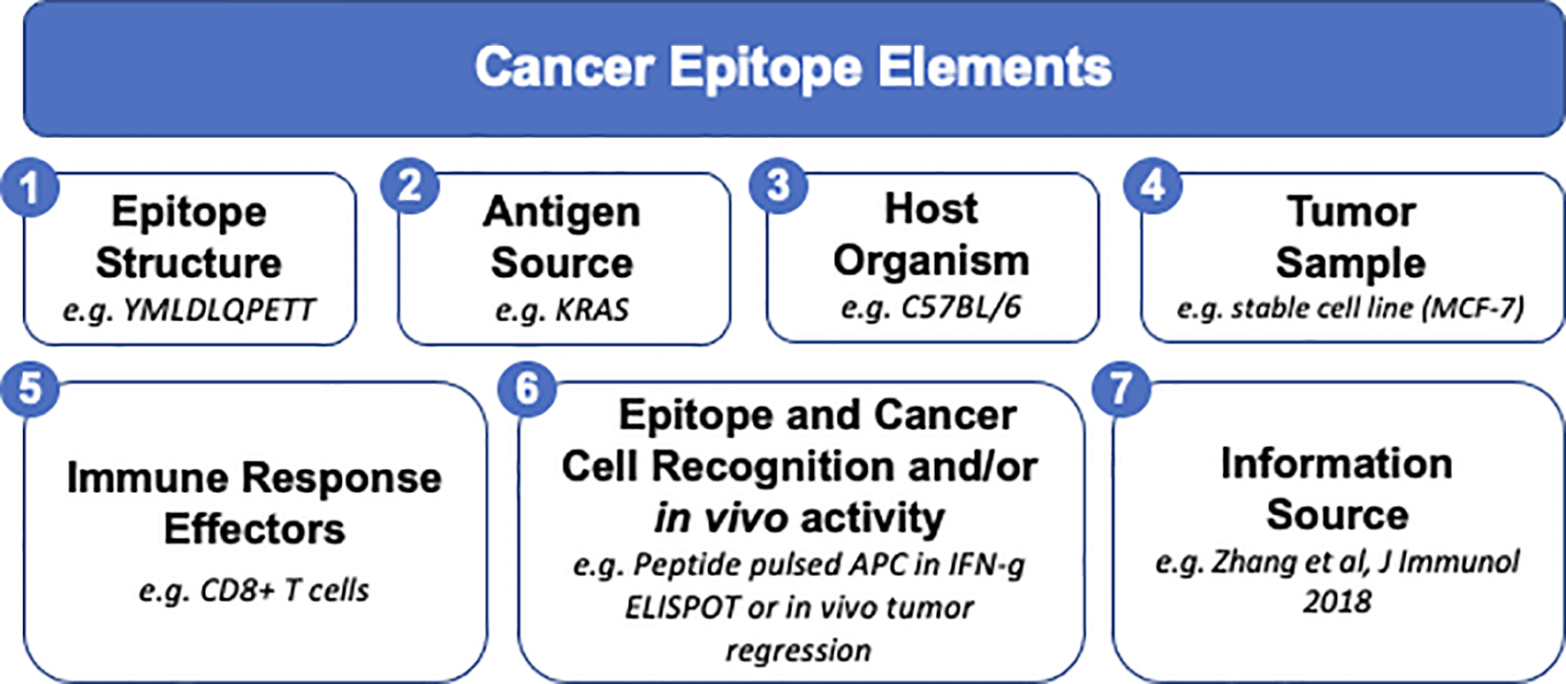
Summary of salient characteristics of cancer epitope data and metadata for CEDAR.

Following initial interviews with cancer experts, we identified the elements relating to an epitope that should be captured in CEDAR (**Figure 1**), including seven main “field groups”, namely (1) related to the structure of the epitope, (2) the protein/antigen source from which the epitope is derived, (3) the host associated with the identified epitope responses, (4) the features of the tumor sample, isolate or model, (5) the effectors of the immune responses (both B and T cell responses), (6) the ability and modality of the effector responses to recognize the epitope and cancer cells, and (7) the source of the information that is captured. CEDAR will also identify whether the information captured is derived from a scientific publication, through a direct submission to CEDAR, or gathered from other online resources, and in each case, clearly state the provenance information.

### Structure and Antigen Source of the Epitope and Type of Cancer Mutations

Different fields and subfields were defined to enable capturing information in a granular yet searchable and accurate fashion. First, we designated fields to capture the amino-acid sequence of the epitope together with associated post-translational modifications such as phosphorylation and glycosylation. In the case of non-peptidic epitopes, such as, for example, CHO epitopes recognized by antibody responses or ceramides used to expand natural killer (NK) T cells, the structures are captured following the format of ChEBI (12) and PubChem (13) resources.

We next defined a set of fields to indicate the general characteristics of the antigen. Specific fields distinguish and classify mutated epitopes (neoepitopes), tumor-associated antigens (TAA) such as differentiation or tissue-specific antigens (e.g., Melan-A, PSA), overexpressed antigens (e.g., HER-2, Muc-1), or cancer-germline antigens (e.g., MAGE, NY-ESO1). For peptidic antigens encoded in the host genome, we defined subfields to capture the gene and protein names of the unmodified antigen, the type [e.g., a self-protein or endogenous retroelement antigens such as long terminal repeats (LTR) or endogenous retroviruses (ERVs) (14)], and its frequency and magnitude of expression in healthy tissues for different tissue and cell types, as well as developmental stages (15). For non-peptidic self-antigens such as carbohydrates or gangliosides, we defined fields to record their presence in different tissues. Similarly, for epitopes derived from non-self-tumor-associated antigens, specific antigens are captured (e.g., protein from HPV). We designated a final set of fields to capture normal properties associated with the antigen, such as subcellular location and involvement in biological functions based on GeneOntology (16, 17), and whether the antigen is a driver gene, known to be causally linked to cancer progression (i.e., oncogene, tumor suppressor gene). A set of subfields also captures expression in pre-malignancies and the frequency and magnitude of expression in various tumor types (18, 19) and cancer cell lines (20).

We designated a distinct but equally important set of fields to capture the type of cancer mutations in the source antigen and their impact on the antigen, including the mutation type, such as single or multi nucleotide variants, frameshift, or non-frameshift indels and chromosomal rearrangements. The effect of the mutation (coding: missense or premature stop codon, frameshift, synonymous; non-coding: splice sites, UTR or other), and the outcome of the mutation on the antigen, distinguishing dysregulated expression, functional impact of the mutation on source antigen (21, 22), structural localization of mutation impact (23), localization in functional domains (21, 24), and known or predicted surface accessibility (23) are captured in additional subfields.

### Fields Related to the Host Organism

We designated a set of fields and subfields to capture the organism associated with the epitope response in terms of species (most references will either be related to human responses or tumor animal models, primarily mice or rats), age, sex, strain or ethnicity, and the major histocompatibility complex (MHC). A separate set of fields was defined to capture the general feature of the cancer, such as natural occurrence and known associated risk factors *versus* induced cancers (genetically engineered organism with spontaneous tumor, xenograft, cancerogenic treatment induced). Cancer classification and diagnosis are captured in designated subfields as well, including anatomical site, histology, tumor stage, and any type of pre-treatment. Additional subfields capture relevant characteristics of the host, such as microsatellite instability (MSI) and HPV status. If the subject from which the responses were derived was vaccinated, the specifics of such treatment are captured in terms of the vaccine antigen delivery format (synthetic peptide, mRNA, DNA plasmid, viral vector, and so on), adjuvant used, administration specifics, and formulation details. Additional fields were designated to capture other types of immunotherapies such as adoptive cell therapy (tumor-infiltrating lymphocytes (TIL) therapy, engineered TCR therapy, chimeric antigen receptor (CAR) T cell therapy, natural killer (NK) cell therapy), and checkpoint blockade therapy (e.g., anti-PD-1, anti-CTLA-4 therapy). If available, doses and dose sizes, information about targeted antigens, corresponding TCR sequences, 3D structures, and therapeutic interventions such as treatments with chemotherapy, radiation, surgery, or oncolytic viruses can be captured as well. Defined subfields will also capture clinical outcome, such as complete response, partial response, or cancer progression, and overall or progression-free survival, as well as reduction of tumor burden, change in tumor markers, and any adverse events of therapy, including autoimmune reactions.

We also designated fields to document the sample, isolate, or model associated with the source antigen of the epitope. Specifically, the sample nature (primary sample/short-term line vs. stable cell line), its occurrence (primary, metastasis, recurring), and whether the sample was obtained pre- or post-treatment. If available, tumor sample purity is also captured (from histology or predicted from sequencing data), as well as the overall mutational burden of the sample. Any available evidence for epitope/antigen expression in terms of frequency and magnitude of epitope/antigen expression in the sample is also documented. Importantly, CEDAR has designated fields to capture the evidence type for the epitope/antigen as detected in whole-genome, whole-exome, transcriptome, or targeted gene panel sequencing, together with the depth and coverage at the epitope site. In the event of a mutated antigen, details related to the mutation are stored, such as its origin (somatic/germline), tools that reported the mutation, read depth at the mutation site, and variant allele frequencies in the tumor DNA sample and RNA sample, if available. Supporting mass-spectrometry elution data are also be captured if available. A separate set of fields was defined to document features related to the tumor environment, including the presence of T cells and characterized subsets.

CEDAR will also include results from *in vivo* experiments such as tumor rejection and/or tumor control data. In such cases, details about the used mouse models or the patients from clinical trials will also be captured.

### Fields Related to Capturing Immune Responses

CEDAR aims to capture the general features of the effector material, including the source of effector cells or antibodies, whether they were (*ex vivo*) T cells, short-term cultured or stable cell lines that were isolated from a tumor-affected host, or whether they were induced/engineered cell lines. Information related to antibody class and subclass and cell phenotypes, including CD4/CD8/NKT subset data and expression of phenotypic markers, is also captured. If available, corresponding TCR and antibody sequences, as well as 3D structures, will also be documented, considering the different levels of resolution associated with various techniques such as targeted sequencing of CDR3 regions and full-length TCR sequencing. We also designated subfields for possible synonymous TCR or BCR sequences encoded by different V(D)J sequences, with the opportunity to capture evidence of immunoediting or antigen loss, if available.

In addition to this, CEDAR also documents the specific assays performed to measure recognition. Examples include ELISPOT, intracellular cytokine staining (ICS) or tetramer assays for T cells, ELISA, antibody-dependent cell-mediated cytotoxicity (ADCC), and fluorescence assays for antibodies. A separate series of fields were defined to capture the effector mode of recognition, namely the capacity to recognize tumor cells directly, cell lines transfected with RNA, or cell lines pulsed with peptides. Particularly relevant for MHC class II-restricted responses is the curation of the type of antigen-presenting cell (APC) involved in the assay determination. A final and most crucial set of fields was defined to capture the results of the assessment, as available in qualitative (positive/negative) and quantitative (magnitude) terms. Importantly, the quality of negative controls associated with the assay, such as data related to MHC and antigen expression, will be carefully curated because a negative result is not valid if the MHC or antigen is not expressed. CEDAR will also capture the number of subjects tested/responded, the type of tested ‘target’, and in the case of mutated epitopes, whether both mutated and wildtype peptides were tested, and the associated outcomes.

### Mapping Database Fields to Community-Supported Standards/Ontologies

In our planning and blueprint development for CEDAR, we have drawn on our experience operating the Immune Epitope Database (IEDB). Our extensive experience with the IEDB, which we initiated in 2003 and have been maintaining and enhancing over the past 18 years, has provided us with important lessons on what to do and, more importantly, what not to do when designing and maintaining an epitope database and analysis resource. By multiple metrics, the IEDB is a success, with >4,000,000 experiments characterizing >1,300,000 epitopes from >22,000 references curated; a monthly rate of >30,000 unique visitors, and over >3,900 citations per year (based on 2020 data). Importantly, even though the IEDB is currently not funded to respond to the needs of the cancer community, up to one-third of current IEDB users are applying its functionality in a cancer research setting. As part of our outreach activities, we have gathered requests from these users on how the IEDB could be improved for cancer researchers.

**Figure 2:**
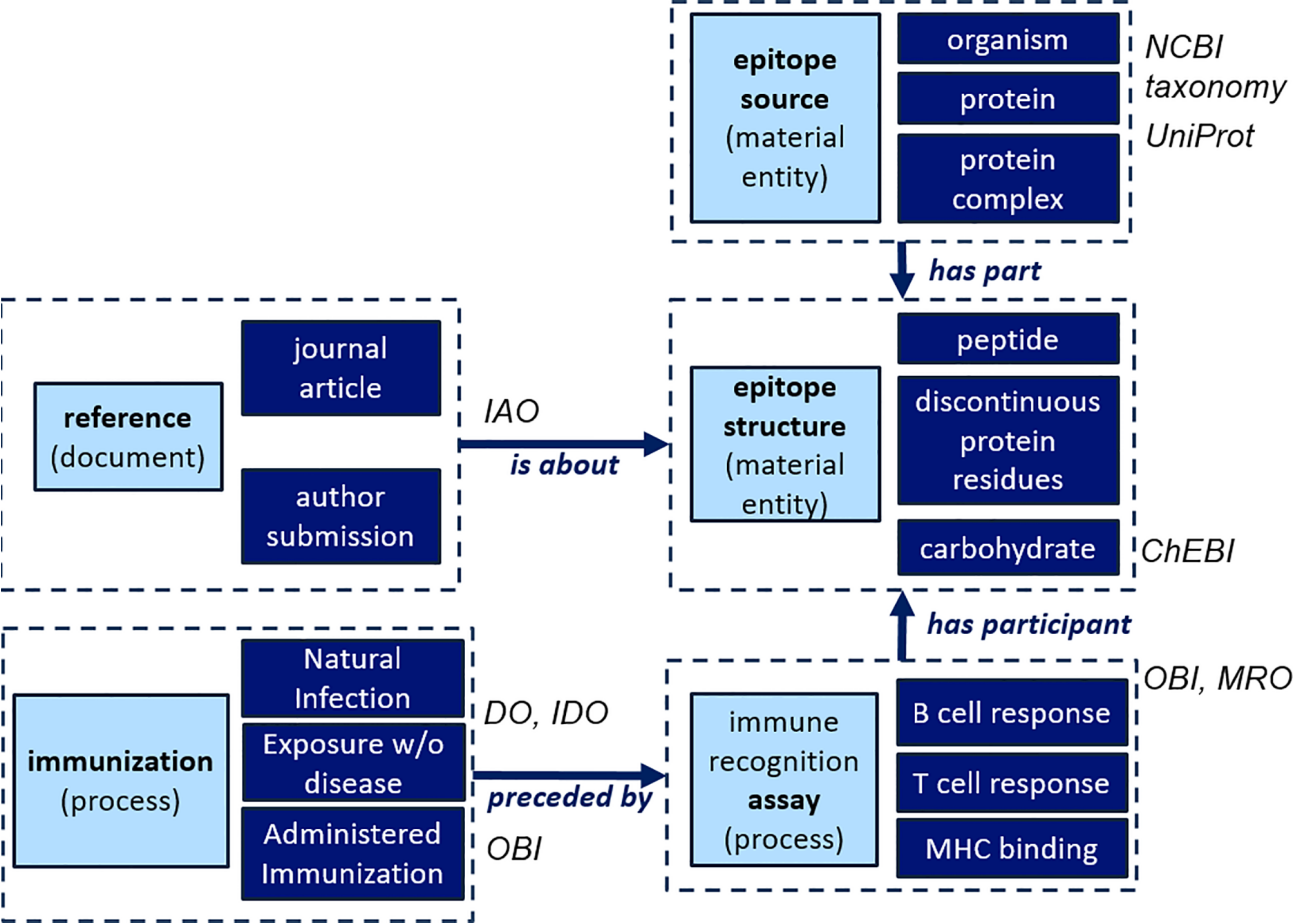
IEDB high-level structure and ontological backbone.

To accurately represent epitope information, the IEDB has developed a semantically well-defined data structure, which utilizes community-supported ontologies for most of its specific fields (**Figure 2**). The core of this data structure has proven to be remarkably flexible and robust, as it has been used to capture over 4 million assay records to date, enabling powerful aggregate queries on epitope information gathered in diverse settings. For example, for epitopes derived from viruses, the NCBI taxonomy is used to capture the particular virus that the epitope is known to originate from. This enables us to capture all synonymous names used to refer to that particular entity (“Human Papillomavirus 16” or “HPV16” or “Human Papillomavirus type 16”). It also allows storing and querying for information at different levels of granularity, such as obtaining all epitopes derived from viruses in the genus “Alphapapillomavirus” or specifying that an epitope was found in a particular isolate of HPV16. As other knowledge resources use the same NCBI taxonomic framework to represent organisms, it makes our data FAIR (findable, accessible, interoperable, and reusable) (25), which is particularly important for the (re-)use of IEDB data by the broader science community (26).

We plan to follow the same paradigm in CEDAR and will ensure that each database field can be mapped to an accessible, community-supported ontology. For fields where the scope overlaps with the IEDB, the same standards can simply be re-used. For database fields that are specific to cancer, standards/ontologies will need to be identified to curate them accurately. We have already identified the need for additional cancer-specific disease terms, including disease states and stages. Disease states will continue to be described using Disease Ontology (DO) (27) terms, which will be expanded and refined to include all cancer terms. Additionally, all cancer-related disease terms will be grouped under the parent term ‘neoplasm’, which aligns with the classification of cancers in the National Cancer Institute Thesaurus (NCIT) (28). Similarly, the NCIT terms will be used to specify cancer stages and link these terms to their official NCIT definitions and identifiers. Our team is proficient in working with vocabulary providers and standardization efforts, and we will enthusiastically embrace recommendations and/or participate in efforts to develop data standards within the ITCR and general cancer research community.

### Development and Implementation of a Web-Enabled Query and Reporting Interface

**Figure 3:**
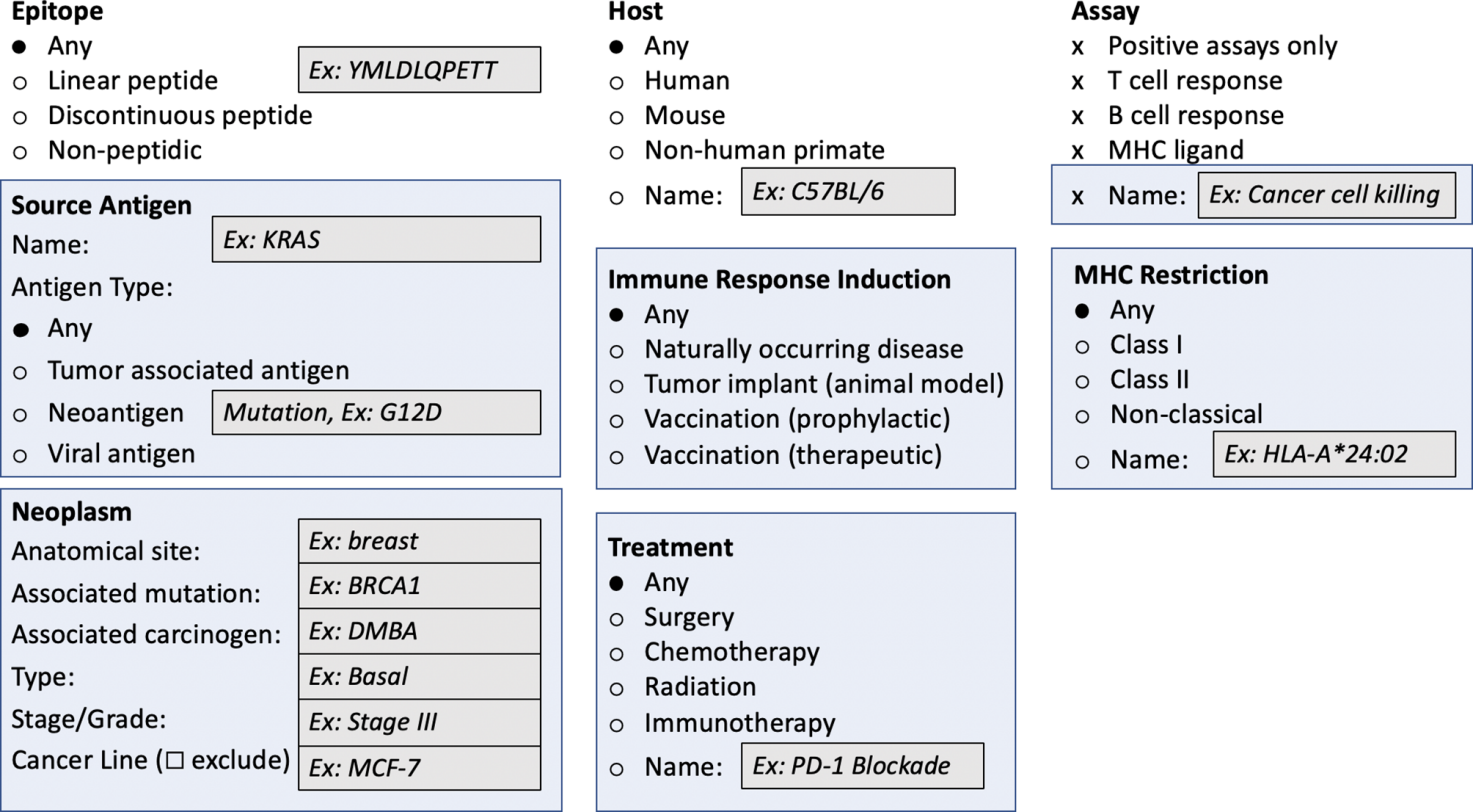
Draft of cancer-specific query interface for CEDAR web portal. Highlighted in light blue are areas that include cancer-specific search parameters not present in the current IEDB interface.

One of the challenges for biomedical community databases is to ensure that the query interfaces are intuitive and that the generated reports provide understandable and scientifically accurate results. An initial design for the CEDAR web query interface (**Figure 3**) focuses on making the most requested pieces of information immediately accessible. This query interface shares fields with those present in the IEDB for epitope structure, host, assays used to characterize the response, and MHC-restriction. At the same time, it enables the direct query for the source of the epitope as it is relevant to cancer, namely source antigen, neoplasm, immune response induction, and treatment. We anticipate adding antigen subtypes, a characterization of the neoplasm/tumor, the ability to select methods used to induce immune responses, and information on the treatment a host was undergoing. It will, for example, be possible to search for all epitopes in a given cancer type or epitopes associated with a specific mutation or gene of interest. The granular curation of the data and the flexible query structure of CEDAR will allow for example to retrieve data related to either natural presentation, recognition of synthetic antigens or both. More detailed searches will also be possible, such as searching for a specific type of assay or for instances where a specific type of treatment occurred.

### Background on the IEDB Curation Process

**Figure 4:**
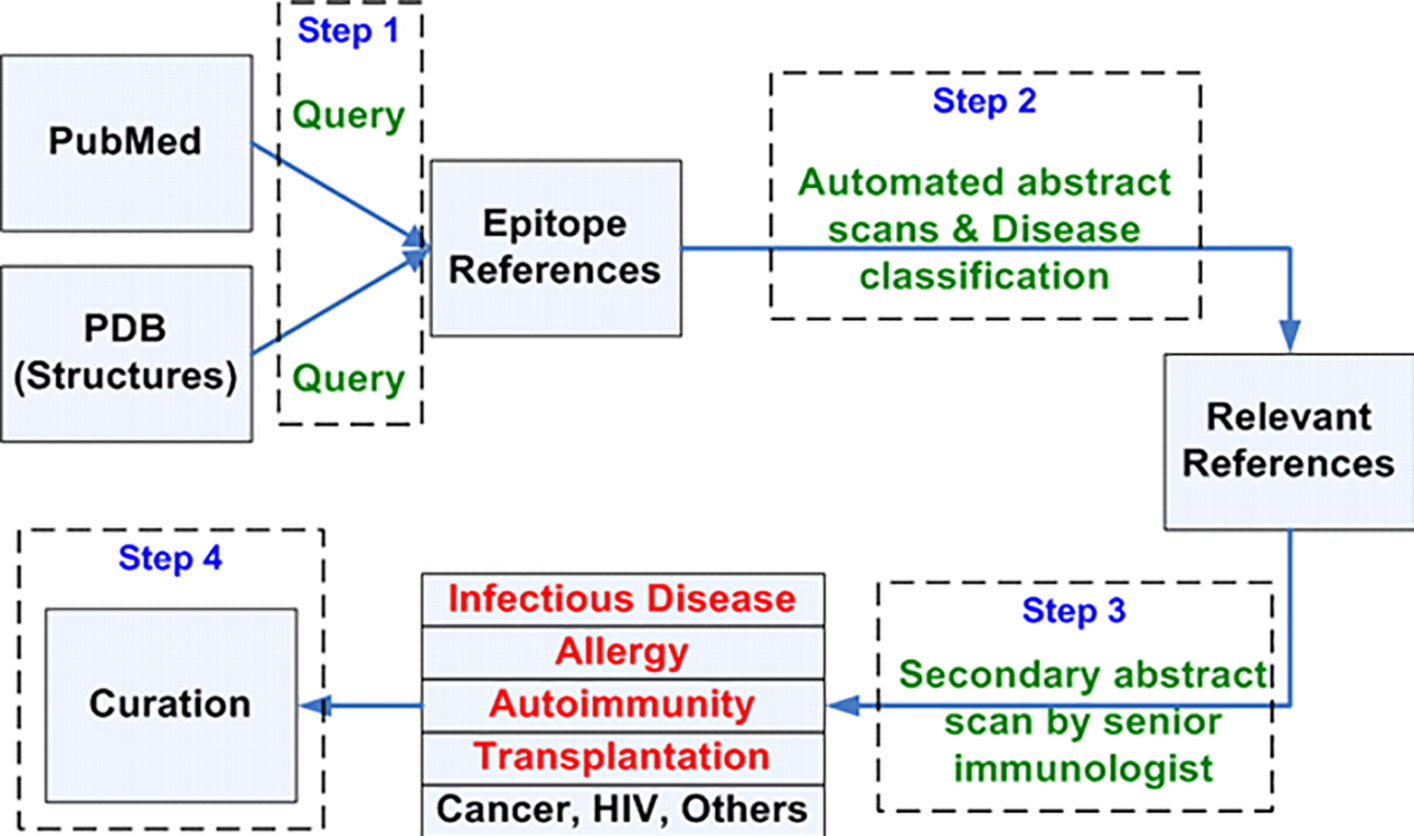
Workflow for identifying curatable journal articles.

To identify and curate relevant publications that contain experimental cancer epitope data, CEDAR will utilize the validated curation approach established and optimized for the IEDB and modify specific steps where required. Over the last 18 years, we have developed, implemented, and continuously optimized the process to identify relevant journal articles for the IEDB and extract information from them, as outlined in **Figure 4**. Scientific literature is constantly monitored by querying the PubMed database on a biweekly basis with broad keyword queries, purposely designed to be comprehensive, in order to retrieve a broad universe of papers that should include all references describing immune epitopes. Over time, these specialized, broad queries have selected over 244,000 references from over 32 million papers available in PubMed.

Based on the abstract, automated text classifier tools and human experts then narrow these references down to those with likely relevance (29–31). The criteria for passing this initial selection require that the reference is within the scope of the database, provide novel data (for example, review papers and use of epitopes as a mere marker or tag are excluded), and describe the epitope molecular structure in sufficient detail and granularity (reports of simple reactivity against whole proteins or undefined structures are excluded). Following these determinations, the reference is classified as “relevant”, and further subdivided into a specific disease category. The full text of relevant articles is then retrieved and assigned to a doctoral-level curator who extracts the data and enters it into the IEDB database curation system. The curated records are peer-reviewed, and once accepted, become visible to the public. The general curation processes are described in detail in previous publications (30, 32–34) and have been continuously adapted as new assay types are established, as has been done to capture receptor data from high throughput sequencing (35).

### Development of a Prioritized Queue of Cancer-Related Articles for Curation

**Figure 5:**
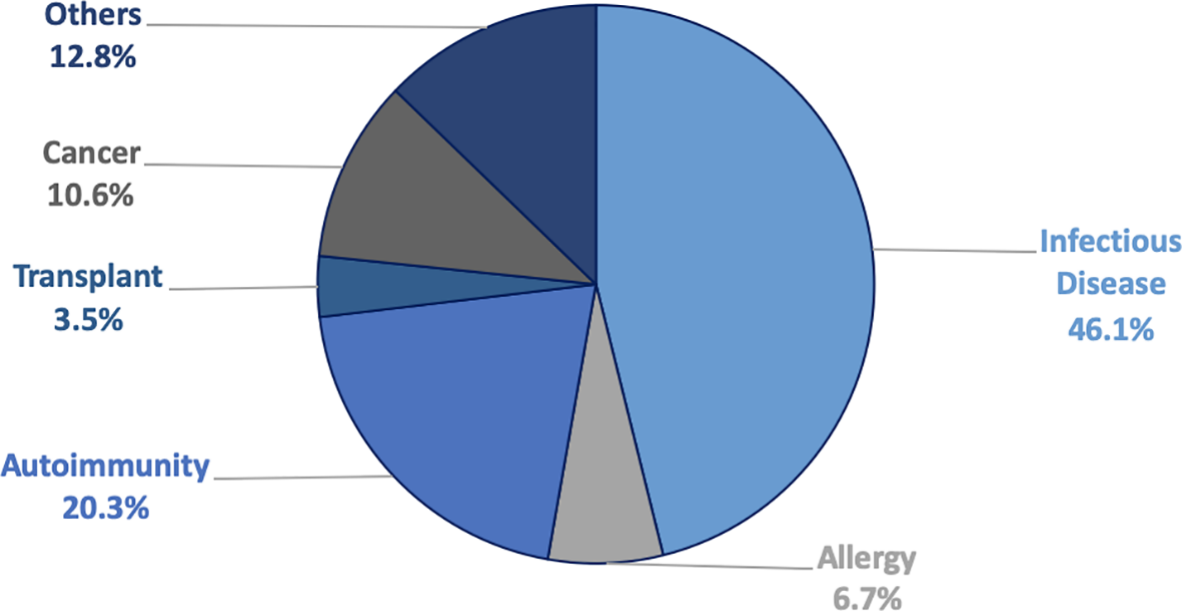
Breakdown of classified and curatable references.

In preparation for cancer curation, papers that contain curatable epitope information as part of the IEDB curation workflow were further categorized by the use of automated text classifiers (29–31, 36) and manual inspection, in broad primary classes (Cancer, Infectious Diseases (excluding HIV which is curated by the Los Alamos database), Allergy, Autoimmunity, Transplantation and “Other”. The percentage of references classified in each of these broad categories is shown in **Figure 5**. Cancer references account for 10.6% of all identified and curatable epitope references. These references were further subdivided into a set of 20 subcategories, grouped as a function of similar antigens and/or cancer types. The most frequent category is melanoma antigens (MAA, 20%), reflecting the prominence of these antigen types in immunological investigations. Other frequent categories are carbohydrate antigens such as Lewis and related antigens (LEWIS, 3.3%), and popular antigens such as mucin (5.5%), Her2 and associated antigens (6.4%), MAGE and associated antigens (4.8%), prostate associated antigens (PROS, 4.0%), p53 (2.6%), antigens associated with lymphoid cancers (LEU, 5.6%), and CEA (2.1%). Neoepitope references were classified separately and presently account for only 5.0%; however, the number of papers in this category has been rapidly rising in recent years.

In addition to this, we plan to inspect and broaden the initial PubMed query by adding keywords to ensure we capture all cancer epitope-specific articles. Our automated document classifier will be re-trained to specifically identify articles that contain cancer epitope-specific information, as we have done for other categories before. Different categories will be addressed in a sequential fashion. Our current first priority for curation includes neoepitopes and T cell epitopes associated with melanoma, breast, and prostate cancers, as these are among the most frequently studied in basic investigations and clinical trial settings.

### Curating Previously Identified Relevant Cancer Articles With Immune Epitope Data

To begin curation of cancer epitope literature, curators will follow the curation rules encoded in the IEDB curation manual, a living document (37), which will be expanded and customized for CEDAR. In brief, for each epitope entered into the database, the structure of the epitope, i.e., an amino acid sequence for peptidic epitopes and a chemical structure for non-peptidic epitopes, is described. If the epitope is naturally occurring, the protein and organism from which the epitope was derived are also entered; for example, the human melanoma antigen recognized by T cells 1 (MART-1) protein. Additionally, all experimental assays in which the epitope was studied are added as T cell, B cell, or MHC ligand assays. The details of each assay include information such as the host, whose immune response was studied, the disease state and stage of the individual, the type of effector cells (CD8+ T cells) or antibodies (monoclonal IgG1) being studied, and the assay method (ELISA, flow cytometry, etc.) that was utilized. Curation also captures the sequences of the epitope-specific TCRs and BCRs.

Curators capture data by entering it into dynamic and interactive web forms designed to optimize productivity and to ensure accurate and consistent data entry. This curation interface enforces curation rules as the curator enters the data, which takes advantage of the ontology-based data structure on a per-field basis. Once the curator has completed entering the data, additional validation rules that cross-compare the content of different fields are checked by the system prior to allowing the curation to be submitted. Just as development will be required on front-end user interfaces to support cancer-specific query and reporting better, the back-end curation system will also require development to allow for appropriate data entry. This system will be updated in coordination with the query and reporting interface development described above and based on the outreach feedback described below.

### Curated Cancer Epitope Datasets for Benchmarking Epitope Prediction Tools

The following sections describe the benchmarking, improvement and development of epitope prediction methods. The results epitope predictions will lead to validation experiments determining which epitopes are actually of biological significance, which is arguably the ultimate goal. These results will, in a recursive modality, be fed back into training of epitope predictions, leading to increased prediction accuracy and significance.

Multiple computational tools and pipelines have been developed to predict cancer epitopes in the scientific community (38). The comprehensive sets of epitopes curated in CEDAR can be used to evaluate the performance of these tools. These benchmarking results will inform tool developers on the most valuable prediction approaches and tool users on which tools they can most rely on. Moreover, the epitope datasets created in this process will be valuable for the broader community in developing new tools. Since many of the tools evaluated will have been trained on subsets of existing data, ‘live benchmarks’ will also be implemented, which consist of automated pipelines that run predictions on epitope datasets just before they are released in CEDAR. We have previously implemented such ‘live-benchmarks’ for MHC class I (39) and MHC class II (40) binding predictions in the IEDB, and the framework established for these is easily expandable for CEDAR.

We previously performed a small benchmark on the predictability of cancer T cell epitopes with different prediction approaches (41). More comprehensive studies can be performed by taking advantage of the curation activities described above, which will already have translated the free text information from journal articles into a structured format. The granular curation in CEDAR will allow to distinguish different datasets, such as peptides shown to *i)* bind MHC, *ii)* be naturally processed and presented by MHC, *iii)* be recognized by T cells when provided as a synthetic antigen, and *iv)* be recognized by T cells as part of a tumor cell. Providing separate datasets for separate biological questions makes it easier for tool developers and users to communicate what a specific algorithm was trained and evaluated on.

We plan to extract these datasets focusing on high-quality experimental records and will make them accessible in formats that can be easily parsed with commonly utilized machine learning algorithms and data analysis packages. We plan to add columns containing additional factors that can help in the predictions. For example, based on the tumor type, the expression level of different source antigens can be estimated using National Cancer Institute (NCI) databases such as cBioPortal (19, 42) and the GDC Data Portal (43), even if that expression data is not specifically measured in the original experiments.

### Development of Novel Tools to Predict Cancer Epitopes

While most methods for predicting cancer T cell epitopes evolve around MHC binding prediction, which is a necessary step for an epitope to be recognized by T cells, other factors, such as the abundance of the epitope (or its precursors) in the tumor and the availability of a TCR repertoire capable of recognizing the epitope, influence T cell recognition. A thorough assessment of the importance of these different features is required, and CEDAR will provide independent datasets continuously acquired over time through the above-described curation process. Here we describe features that have been considered by multiple investigators as drivers of differential immune recognition (11, 44–49).

**Figure 6:**
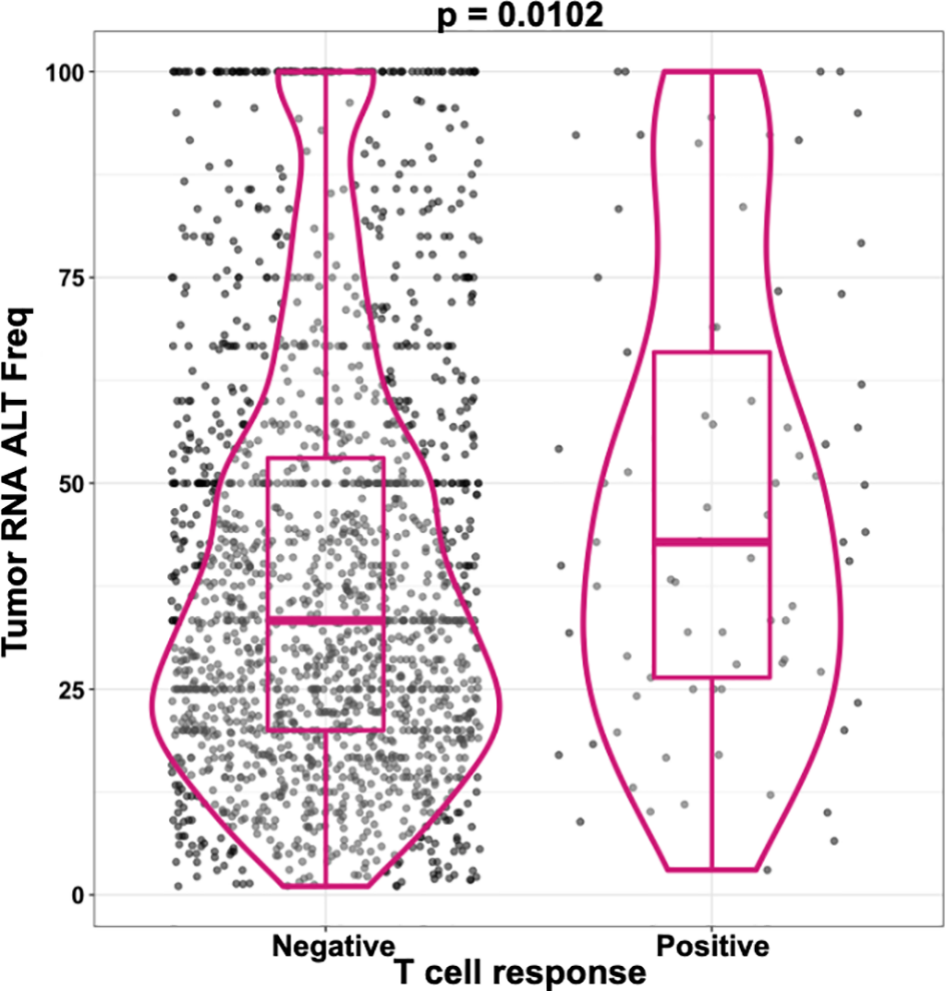
RNA correlation with neoantigen recognition.

We and others have performed analyses correlating measures of the abundance of an MHC ligand with its likelihood to be recognized by T cells (11, 46–48, 50). For cancer epitopes that arise from a mutation (neoepitopes), the abundance is expected to correlate with the frequency of the mutation in the tumor DNA, as well as with the RNA expression level. Our preliminary analysis of in-house data, as well as data recently published from the NCI (46), showed that the variant allele frequency in the RNA is significantly correlated with neoantigen recognition (**Figure 6**). Thus, including a measure of epitope abundance into machine learning methods is expected to improve cancer epitope prediction. Accordingly, for non-mutated cancer epitopes, the abundance of the associated source antigen, for example as measured by RNA-Seq or proteomic analysis, might improve epitope prediction and will be analyzed in detail.

The TCR repertoire is shaped by both central and peripheral tolerance. Specifically, T cells with receptors binding to self-peptides are expected to undergo apoptosis or adopt a regulatory phenotype. Thus, we and others have hypothesized that peptides with high similarity to host peptides have a lower likelihood to be recognized by T cells (44, 49, 51, 52). For cancer epitopes, the similarity to self-peptides is expected to be of particular relevance, given that - by definition - cancer epitopes are highly similar to host peptides. It will be important to develop metrics of peptide similarity that correlate best with peptide immunogenicity in a cancer epitope setting and determine if they improve the performance of epitope immunogenicity predictions (53). Furthermore, it has been hypothesized that, as TCRs have evolved to be cross-reactive for similar epitopes in order to provide protection from rapidly evolving pathogens (54, 55), cancer epitopes with similarity to pathogen sequences may be more immunogenic, and this similarity may correlate with clinical outcome (56). It was also suggested that neoantigens from driver genes are more likely to be recognized by T cells (46).

As entries in CEDAR will be linked to specialized databases that host such information, we will be able to easily access all information and include it in the training of machine learning methods. The Cancer Genome Atlas (TCGA), the Catalogue Of Somatic Mutations In Cancer (COSMIC) (57), and the Cancer Gene Census (CGC) (57) are all examples of databases that can be utilized to retrieve information about recurrent cancer mutations and whether a mutation is affecting a driver gene or not. Newly generated sets of experimentally validated T cell epitopes that will become available in CEDAR will allow users to assess specific hypotheses, such as mentioned above and *in silico* prediction pipelines in general, that were created and tested on limited datasets.

**Figure 7:**
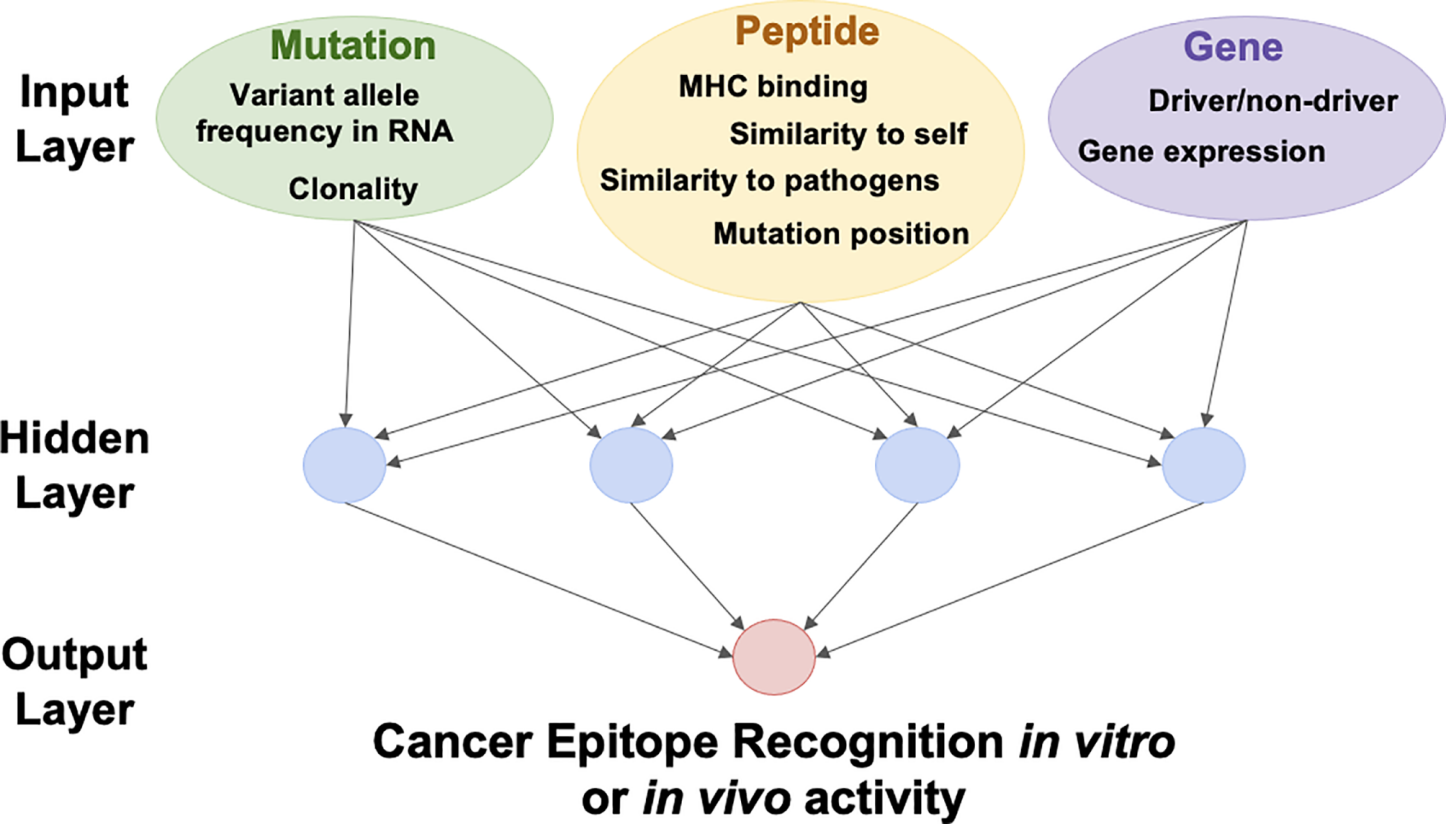
Schematic of an Artificial Neural Network we could implement to learn determinants of Cancer Epitope Recognition.

Using the newly curated datasets from CEDAR, different combinations of features can be included in training machine learning methods to optimize the prediction of epitope recognition (**Figure 7**). The model can be trained to predict any cancer-epitope related outcome, such as cancer epitope recognition *in vitro* or *in vivo activity* (such as tumor regression or experimental model outcomes). With more epitope data becoming available, we will regularly update classifiers and assess whether the data contains additional features (including specificity to TCRs) that might be of relevance for predicting cancer epitopes. We estimate that the size of the training data set made available through the CEDAR curation of approximately 1,770 references will equal at least 50,000 epitopes, based on a comparison of the current epitope count in the IEDB. This data set should be sufficiently large to explore multiple training strategies and features for consideration.

### Development of Cancer Epitope Analysis Tools

In our interactions with cancer immunologists and clinicians, it was pointed out that immunoinformatics tools to predict MHC binding and antigen-processing are not user-friendly, as they often require elaborate pre- and post-processing of input and output data to make them applicable in the cancer setting. We identified several recurrent operations involved in analyzing cancer epitopes, and we plan to create analysis tools that allow automation and integration into cancer epitope-specific pipelines.

Determining what neo-peptides are generated by a given mutation, for example, is non-trivial when complex mutations such as frameshifts or splicing variants are involved. We plan to provide tools to generate lists of overlapping n-mers to be included in experiments, given a mutation of interest (e.g., KRAS G12V or chr:12 341234 A<T).

It is also of interest to identify if a given mutation or peptide has already been tested for immunogenicity, and if so, in what context. CEDAR will be interlinked with specialized databases such as TCGA, COSMIC, the CGC, and dbSNP, as mentioned above. We plan to develop tools to retrieve all available information for a given mutation, including if a given peptide has already been described in CEDAR (as a cancer epitope) or the IEDB (e.g., for pathogen-derived epitopes) and whether it is found elsewhere in the host proteome. Another important analysis tool will provide MHC binding predictions for a set of mutated and associated wildtype sequences in the context of a set of MHC alleles.

Likewise, it is of interest to determine if TCR or BCR sequences have been described before. For CEDAR, all published cancer-specific receptor sequences and their recognized cancer epitopes will be curated. This combined database will provide a comprehensive list of receptor sequences and the epitopes they recognize. We have developed a ‘receptor lookup’ tool (58), which accepts the TCR β chain CDR3 sequence as an input, and identifies if TCRs with that exact sequence (or a highly similar one) have been previously characterized, and if so, what the previously identified epitope specificity is. This tool was designed to handle large input datasets, such as those generated by TCR repertoire sequencing experiments, and will annotate for each receptor if it has been found before and what epitopes it was previously reported to recognize in both cancer and other disease settings.

## Discussion

Here we present our vision and “blueprint” to design and implement the Cancer Epitope Database and Analysis Resource (CEDAR), which will provide a comprehensive collection of cancer epitopes curated from the literature, as well as cancer epitope prediction and analysis tools. CEDAR will leverage our decades of experience from the development of the IEDB, which is fully operational and has been funded since 2003 through a contract from NIAID, with an extension to 2025. The IEDB focuses on allergy, infectious disease, transplantation, and autoimmunity but excludes cancer. Of note, the current **Figures 1**–**3** reflect the initial prototype based on the direct result of the input received in the initial planning stages by our panel of experts. We however expect that these will evolve over time as the prototypes are implemented and additional feedback is received.

It is now well recognized that understanding the nature of cancer epitopes and their cognate receptors enables us to answer important scientific questions. For example, researchers are examining how the mutation and epitope load in a given tumor relate to the success of checkpoint blockade treatments (4). In addition to this, current research explores epitope-based vaccines and the transfer of epitope-specific T cells and TCRs for use in personalized therapies (4, 5, 59, 60). Epitopes recognized across different individuals provide ideal targets for more cost-effective, off-the-shelf immunotherapies, re-igniting interest in tumor-associated antigens. While mutation-based neoantigens have received considerable attention in recent years, the CEDAR initiative will also curate all data related to cancer-specific but non-mutated antigens, e.g. based on cancer-specific protein expression and processing variations, or cryptic antigens.

This interest is not limited to T cells, as several therapies also take advantage of defined antibodies and BCRs. Moreover, the ability to readily sequence TCRs and BCRs through single-cell sequencing studies of tumor tissues has provided an impetus to develop tools that facilitate the identification of tumor-specific T cells and B cells in these samples. To address these needs, CEDAR will provide a central, freely accessible catalog of cancer epitope and receptor data linked to the biological, immunological, and clinical contexts in which they were described. The ultimate goal is to come “full circle” and link epitope recognition and immunological readouts to outcomes of disease, treatment, and vaccination. We also aim to use these data to develop and evaluate machine learning-based epitope and TCR/BCR specificity prediction tools for the analysis resource component of CEDAR.

The CEDAR website will initially be developed based on our experience in translational cancer research, as well as feedback obtained from a diverse set of cancer experts. The website will enable intuitive and scientifically accurate cancer-specific queries and reports. This will be implemented by leveraging the existing IEDB database, curation, and query and reporting infrastructure, and expanding it to represent clinical and disease phenotypes beyond what is currently in the IEDB. Additional fields relevant to cancer will be accurately captured, such as different forms and histologies of cancer and associated immunological, biological, and clinical information. Based on our preliminary data, the modifications required in the IEDB infrastructure to enable CEDAR can be implemented in a period of 12 months. Once established, subsequent modifications to CEDAR will be driven by broader community feedback.

Curation of immune epitope data from literature, relevant to cancer immunology, will include B and T cell epitopes associated with cancer antigens, and in particular, naturally processed and presented epitopes recognized in the context of a tumor, such as the ones recognized by tumor-infiltrating lymphocytes. Epitope data gathered in immunotherapy studies, in human clinical trials and animal models, will also be captured along with the sequences of both naturally occurring and engineered cancer epitope-specific TCRs and BCRs.

Data related to cancer-specific HLA ligandomics analysis by mass spectrometry will also be prominently curated and displayed, as well as data demonstrating epitopes’ natural presentation on tumor cells. Currently, natural ligand data is already curated in the IEDB, and more than 872,001 eluted ligands are curated and accessible through the IEDB website. These data together with any cancer-specific data will be accessible through both the IEDB and CEDAR webpages.

The granular curation of the data and the flexible query structure of CEDAR will allow the user to extract the data most relevant for different queries. For example data related to natural presentation or recognition of tumor targets is arguably the most important whenever available, whereas recognition of synthetic antigens by T cells has frequently led to false positive results. The flexible query structure of CEDAR will allow to retrieve data related to either natural presentation, recognition of synthetic antigens or both.

CEDAR will curate all cancer epitope data obtained either *in vivo* or *in vitro*. Tumor rejection or tumor control data is one of the measures of activity of cancer epitopes and will be curated as such where available. Indeed, several studies have published data in mouse models and human clinical trials where the ability of individual cancer epitopes has been tested *in vivo* (61–65). Arguably, this is the most significant activity of a cancer epitope. A number of studies also previously reported T cell responses against cancer epitopes *in vitro*, which however did not result in clinical activity when tested *in vivo* (66–68). Furthermore, human studies (69, 70) and mouse studies (71, 72) have highlighted contradictions in the data on neoepitope recognition. As CEDAR will include data from both, *in vitro* and *in vivo* experiments, it will be possible to analyze any correlations between T and B cell responses *in vitro* and associated antitumor efficacy *in vivo.*

To the best of our knowledge, CEDAR would provide the first comprehensive and curated cancer epitope database in which the biological, immunological, and clinical context is captured with high granularity and is retrievable for analysis with ease and accuracy. Currently, none of the available repositories capture comprehensive cancer epitope information with the necessary granularity. CEDAR will provide query and reporting strategies specifically designed to meet the needs of cancer researchers to make cancer epitope data and metadata accessible. These strategies are designed specifically for CEDAR and will be developed in a timely and cost-effective manner, relying on the existing IEDB infrastructure, which is based on over 18 years of work experience and development.

Large efforts have been dedicated to developing novel approaches for improved prediction and/or identification of cancer neoepitopes (1, 41, 52, 56, 73–77). Each of these efforts proposed different features to complement HLA binding prediction to improve the ability of identifying cancer neoepitopes. However, these studies are highly heterogeneous in terms of data generation, validation techniques, and the generality of the obtained conclusions, further challenged by an often very limited sample size. The Tumor Neoantigen Selection Alliance (TESLA) has provided an attempt to address these issues by generating uniform data sets to be used by the community for prediction of neoepitope candidates with subsequent experimental validation (49). The main conclusion from this work was that immunogenic tumor epitopes ‘are those tumor peptides that have strong MHC binding affinity and long half-life, are expressed highly and have either low agretopicity or high foreignness’ (49).

CEDAR will further this kind of analysis and provide a validated set of cancer epitope prediction and analysis tools. Users will have access to implementations for published tools that currently have no web-accessible versions and, objective and transparent benchmarks of all tools will be performed using literature data that becomes available in CEDAR through ongoing curation efforts. In line with what has been the case for general T cell epitope prediction tools, the availability of comprehensive datasets within the IEDB and benchmarking has been pivotal for the identification of well-performing tools, excluding anecdotal results. Similarly, we expect that these properties of CEDAR will allow users to identify none-dataset specific properties and help move the field of cancer neoepitope prediction forward. Finally, new tools will be developed based on lessons learned from the benchmarks that include cancer-specific considerations, such as gene expression. Additionally, we aim to provide a tool that will compare the mutant and wildtype sequences in terms of their ability to bind cognate HLA molecules and trigger T cell responses when evaluating immunogenicity.

We will greatly expand the development, hosting, and availability of different strategies to predict the immunogenicity and clinical efficacy of cancer epitopes, as well as their potential as a surrogate marker of positive clinical evolution following cancer treatments. The availability of large, curated cancer epitope datasets, reference implementations of prediction approaches, and clear metrics of success is necessary to inform both the community of tool developers on what makes a tool useful and the community of tool users on which tool to use for their application. Users will be provided with unbiased, objective, and transparent evaluations of different epitope prediction tools side-by-side, with the code being made publicly available. Cross-comparison of prediction approaches on epitope datasets derived from cancer *versus* other diseases (infection, allergy, autoimmunity) will determine if there are predictable features of cancer epitopes that differentiate them from other epitopes.

As the CEDAR data will be hosted side-by-side with IEDB data, the resulting combined dataset will encompass all known epitopes and their TCRs and BCRs, regardless of disease context. This dataset will enable highly innovative analyses, namely the ability to identify TCR and BCR sequences with known (or inferred) epitope specificity from repertoire sequencing data. With the increasing ease of isolating and sequencing TCRs, the identified repertoire can be compared to the continuously growing database of known TCR:epitope:MHC interactions. This will allow identification of TCRs in tumor-associated T cells targeting known neoepitopes or tumor-associated antigens, as well as TCRs targeting viral epitopes (60, 78, 79). Some studies have reported enrichment of TCRs that recognize viral epitopes in TIL that could be cross-reactive, as well as TCRs capable of recognizing unmutated self-peptides expressed in normal tissue (80, 81), which could result in autoimmune side-effects of checkpoint blockade treatments. Ultimately, CEDAR will prove to be a powerful resource for the cancer community and will help advance cancer research and the development of effective cancer therapies.

## Data Availability Statement

The original contributions presented in the study are included in the article/supplementary material. Further inquiries can be directed to the corresponding author.

## Author Contributions

ZK-Y, NB, AS, and BP prepared the manuscript. HC, MN, EZ, DK, JC-G, PR, and SPS reviewed and edited the manuscript. All authors contributed to the article and approved the submitted version.

## Funding

Research reported in this publication was supported by the National Cancer Institute of the National Institutes of Health under award numbers U24CA248138 and U01DE028227.

## Author Disclaimer

The content is solely the responsibility of the authors and does not necessarily represent the official views of the National Institutes of Health.

## Conflict of Interest

The authors declare that the research was conducted in the absence of any commercial or financial relationships that could be construed as a potential conflict of interest.

## Publisher’s Note

All claims expressed in this article are solely those of the authors and do not necessarily represent those of their affiliated organizations, or those of the publisher, the editors and the reviewers. Any product that may be evaluated in this article, or claim that may be made by its manufacturer, is not guaranteed or endorsed by the publisher.
